# Design and Characterization of a Wearable Inertial Measurement Unit

**DOI:** 10.3390/s24165388

**Published:** 2024-08-21

**Authors:** Diego Valdés Tirado, Gonzalo García Carro, Juan C. Alvarez, Antonio M. López, Diego Álvarez

**Affiliations:** Multisensor Systems and Robotics Group (SiMuR), Department of Electrical, Electronic, Computers and Systems Engineering, University of Oviedo, 33203 Gijón, Spainamlopez@uniovi.es (A.M.L.); dalvarez@uniovi.es (D.Á.)

**Keywords:** IMU, gyroscope, accelerometer, error characteristics

## Abstract

The utilization of inertial measurement units as wearable sensors is proliferating across various domains, such as health care, sports, and rehabilitation. This expansion has produced a market of devices tailored to accommodate very specific ranges of operational demands. Simultaneously, this growth is creating opportunities for the development of a new class of devices more oriented towards general-purpose use and capable of capturing both high-frequency signals for short-term, event-driven motion analysis and low-frequency signals for extended monitoring. For such a design, which combines flexibility and low cost, a rigorous evaluation of the device in terms of deviation, noise levels, and precision is essential. This evaluation is crucial for identifying potential improvements and refining the design accordingly, yet it is rarely addressed in the literature. This paper presents the development process of such a device. The results of the design process demonstrate acceptable performance in optimizing energy consumption and storage capacity while highlighting the most critical optimizations needed to advance the device towards the goal of a smart, general-purpose unit for human motion monitoring.

## 1. Introduction

The use of inertial measurement units (IMUs) as wearable sensors to monitor human motion play an important role in a variety of applications, including health monitoring, pedestrian dead reckoning, fall detection, gesture recognition, occupational safety, and sports performance analysis [1]. Novel application domains for these devices are continuously being identified in health care [2], sports [3], rehabilitation [4], and human–computer interaction [5].

Consequently, there has been a substantial increase in the demand for high-precision inertial sensing platforms. But the spectrum of operational requirements that such device must address is exceedingly broad. Therefore, the market has witnessed a substantial proliferation of devices designed to achieve good performance in specific applications [6] or for widespread use by reducing costs [7].

We anticipate that the future expansion of these devices into diverse applications will necessitate a design prioritizing both flexibility and affordability. Such a design would allow the same device to operate at high sampling rates for short periods or at moderate rates over extended time frames. Furthermore, a substantial research gap remains in the metrological characterization and performance evaluation of inertial platforms. Their actual working conditions, such as energy consumption, storage capacity, and noise characterization, are rarely addressed in the existing literature [8].

To address this gap, this study presents a preliminary design of a wearable device of this type. Emphasis is placed on its evaluation and characterization [9,10,11]. The device was assembled, then analyzed from several perspectives, including power consumption, sampling performance, and error characterization. Precise hardware control and rigorous performance characterization are essential prerequisites for future design optimizations in the pursuit of a versatile, affordable, accurate, and robust general-purpose device.

The results show a good response across the three examined areas highlight the most critical optimizations needed to improve the device to achieve the goal of a smart general-use unit for human motion monitoring.

## 2. Materials and Methods

After a series of preliminary tests, a sensor capable of satisfying the best consumption/performance ratio in the context described above was selected. The optimization of components and smaller hardware resources was also started, seeking to increase the density of components on the PCB by means of 0402 resistors and capacitors, suppressing unnecessary sockets, etc.

### 2.1. Description of the Sensor

The LSM6DSL IMU sensor from STMicroelectronics (Geneva, Switzerland) was selected [12] over other well-known IMU sensors such as the MPU6050, BMI270, or ICM-42670-P. This choice was motivated by by several factors. First, eliminating the magnetometer component simplifies the design and reduces potential sources of error. Secondly, the LSM6DSL’s low power consumption makes it particularly suitable for wearable devices, where battery life is a critical consideration. Lastly, the search for a high sampling frequency further supports its selection. All these characteristics are summarized in Table 1, compiled from the datasheets of each sensor [12,13,14,15], demonstrating the suitability of the chosen sensor for the desired application.

### 2.2. Description of the Microcontroller

Choosing the right microcontroller is critical to optimizing both performance and energy efficiency in embedded systems. After a thorough evaluation of various microcontroller families, the STM32L051 emerged as the optimal choice due to its outstanding low power consumption, which is essential for applications where energy efficiency is paramount. Compared to other options, such as the ESP32-S3, AT32UC3, and Atxmega32C4, the STM32L051 has a much lower active power consumption of just 4.4 mA. This is significantly lower than that of its counterparts, ensuring that the system meets performance benchmarks while maintaining energy efficiency. The characteristics of this unit, coupled with its ultra-low idle and sleep power consumption (1.2 mA in idle and 0.29 µA in sleep mode), make it a superior choice over other microcontrollers such as the AVR or ESP families. It also benefits from being available in a pinless package that is only 7 mm wide, which, together with the elimination of the external clock, optimizes the space on the PCB. Table 2, based on microcontroller datasheets [16,17,18,19], provides a detailed comparison of the STM32L051 with other prominent microcontroller families, highlighting its advantages in terms of power efficiency and integrated features.

### 2.3. Description of the Device Design

**Figure 1:**
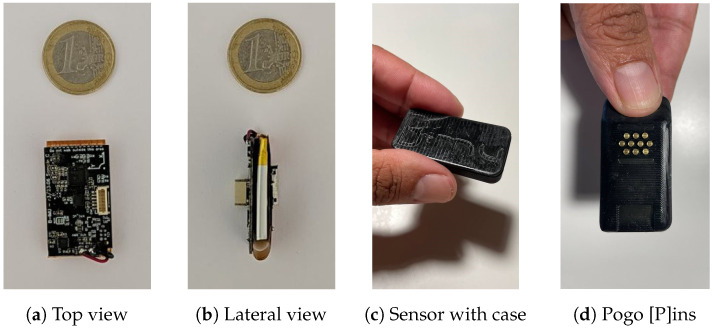
Bimu sensor.

The device design consists of two PCBs placed on both sides of the battery and connected by a flex cable, as can be seen in Figure 1a,b. Only the SD card socket and the contacts for the socket are located on the main side, and most of the system components are located on the other side. This saves space and miniaturizes the entire assembly. The final device with its case is shown in Figure 1c,d.

For battery management, the circuit utilizes an STNS01PUR integrated chip from STMicroelectronics, which combines a battery management system (BMS) for safe charging with a 3.1 V LDO voltage regulator. This chip provides overcharge, over-discharge, and overcurrent protection to prevent the battery from being damaged under fault conditions. Additionally, it features a charger-enable input to stop the charging process when battery overheatingis detected by external circuitry. The charging process is conducted via two of the Pogo pins, designated as GND and +5 V, while the remaining pins are employed for memory reading operations. When shutdown mode is activated, the battery power consumption is minimized to less than 500 nA, maximizing battery life during shelf time or shipping. The battery is a 220 mAh 402030 LiPo with an integrated NTC thermistor. To simplify the device’s operation and eliminate the need for push buttons, we opted to utilize the tilt function of the IMU accelerometer. The test parking functionality (sync SD) is activated by shaking the sensor when it is tilted beyond a specific threshold of 4 g, enhancing the device’s functionality. Additionally, a low-power RGB LED is used to indicate different modes and sensor states through a combination of its three colors, patterns of continuous or intermittent flashing, and fading in and out.

### 2.4. Test Procedures

This section details the test procedures used to validate and assess the proposed system’s performance. We systematically evaluate the solution’s accuracy, reliability, and efficiency, highlighting its potential in scenarios with high data capture rates.

**Figure 2:**
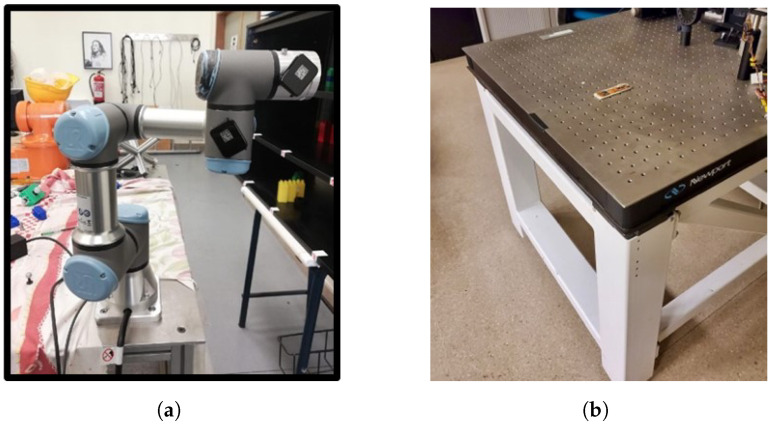
Sensor data characterization. (**a**) Robot for accurate sensor positioning. (**b**) Optical table anti-vibration.

Before obtaining any result, the IMU box alignment matrix is calculated to ensure the accurate orientation of the device axes relative to the box axes within the device. The experiment begins by aligning each box axis with gravity as a consistent reference. This alignment is achieved by placing the device in the end effector of a UR3 robot (Figure 2a). The theoretical acceleration matrix (*A*) represents ideal outputs under perfect alignment, while the measured matrix (*B*) records actual outputs given the current device orientation. The alignment matrix (*R*) is derived as

(1) $$R_{3\times 3}=A_{3\times m}{\left(B_{m\times 3}^{T}\right)}^{-1}$$

**Figure 3:**
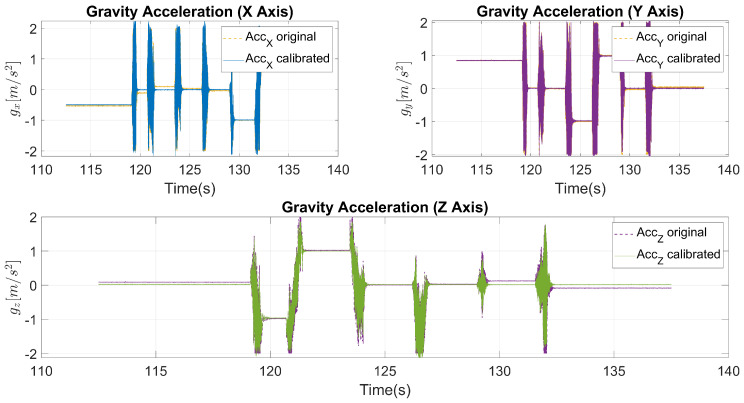
Accelerometer calibration.

This misalignment was quantitatively assessed and corrected using an alignment matrix. As depicted in Figure 3, the original signal exhibited noticeable discrepancies when compared to the corrected signal. After applying the calibration matrix, the corrected signal demonstrated a significant improvement, closely matching the intended orientation and minimizing the observed deviations.

#### 2.4.1. Sampling Frequency

In evaluating the LSM6DSL and STM32L051K8 system, we focused on the constraints of the SPI communication protocol. Using a digital analyzer, we scrutinized the SPI signals between the sensor, microcontroller, and SD memory card. We specifically adjusted the sensor’s sampling frequency at various rates to identify the point at which the SPI buffer became saturated.

The sensor was precisely configured to operate within a measurement range of ±4 g for acceleration and 250 degrees per second (dps) for angular velocity.

#### 2.4.2. Power Analysis

A systematic approach was employed by integrating a 0.1 Ω shunt resistor for precise current measurement. Additionally, an FLIR E60 camera was utilized to analyze temperature variations, enhancing the assessment of thermal dynamics within the system. This setup allowed for the monitoring of charging efficiency and thermal performance under various operational conditions and modes. The documentation and analysis of energy consumption across different states provide a comprehensive understanding of the system’s energy dynamics, facilitating phase optimization.

#### 2.4.3. Standard Deviation

The standard deviation (2) is a statistical measure that quantifies data dispersion and, within the scope of this study, enables the evaluation of consistency and precision of measurements along each axis. The method employed to calculate this parameter involved the sensor collecting data over a 10-min duration. The sensor was situated on a stable, vibration-isolated table (Figure 2b).

(2) $$s=\sqrt{\frac{1}{N-1}\sum_{i=1}^{N}{\left(x_{i}-\bar{x}\right)}^{2}}$$

#### 2.4.4. Drift

Drift refers to any systematic change or undesired variation in sensor measurements over time, occurring independently of external changes in operating conditions. To characterize drift, the sensor was placed on a stable, anti-vibration table for 24 h. Subsequently, a linear regression analysis was applied to estimate the drift along each axis (x, y, and z).

#### 2.4.5. Fixed Bias

The fixed bias represents a consistent error in sensor measurements that is inherent due to its systematic nature. To neutralize this error, measurements from opposite directions are summed, which effectively doubles the fixed bias for each sensor type (3). Samples were collected over 150 s by positioning the device at opposite angles for accelerometer readings along each axis and by applying reverse angular velocities for each axis during gyroscope evaluation using a robot arm, as illustrated in Figure 2a.

(3) $$Bias=\frac{1}{2}\left[s_{x}^{+}+s_{x}^{-},s_{y}^{+}+s_{y}^{-},s_{z}^{+}+s_{z}^{-}\right]$$

#### 2.4.6. Scale Factor

The scale factor measures how the sensor responds to specific measurements within its range. It is calculated by subtracting the averages of two measurements to eliminate fixed bias effects, then doubling the sensor output (4). For accelerometers, the specific reference measurement is local gravity, and for gyroscopes, it is the rotational speed of the device.

(4) $$Scale_{\mathrm{acc}}=\frac{1}{2}\frac{1}{s_{ref}}\left[s_{x}^{+}-s_{x}^{-},s_{y}^{+}-s_{y}^{-},s_{z}^{+}-s_{z}^{-}\right]$$

#### 2.4.7. Power Spectral Density

Following [11], noise characteristics were analyzed using power spectral density. In this analysis, the sensor was kept at rest for ninety minutes during two separate tests. Power spectral density (PSD) quantifies the intensity of unwanted signals across different frequencies, providing insights into the sensor’s response to environmental variations and electronic interferences. This comprehensive analysis is crucial for understanding the sensor’s operational fidelity under real-world conditions.

#### 2.4.8. Allan Deviation

An Allan deviation test was also conducted as described in [11]. The sensor was kept at rest for ninety minutes during two separate tests. The Allan deviation assesses the stability of the sensor’s measurements over time, identifying the types of noise that affect the sensor. This detailed examination is essential for determining the long-term reliability and accuracy of the sensor in various operational scenarios.

## 3. Results

### 3.1. Sampling Frequency

The device was designed to operate at a maximum frequency of 4000 Hz, which establishes its upper limit for data acquisition speed. Each sensor access requires a reading time of approximately 112 μs. This reading duration encompasses the time needed for the sensor to capture the data and make them available for processing.

In addition to the sensor reading time, the microcontroller requires an additional 108 μs to process the data read from the sensors. During this time, the microcontroller performs necessary computations and transmits the processed data to the memory for storage. Thus, the combined cycle of reading and processing totals an average of 220 μs for each operation.

### 3.2. Power Analysis

The power consumption report delineates distinctive patterns across four key phases of device operation, each represented by a specific color code.

During the idle phase (blue), the device exhibits a current consumption of 5.9 mA, which represents the minimum current necessary to maintain the device’s standby functionalities. The file-opening phase (purple) is characterized by a notable increase in power consumption, reaching a peak of 44 mA, which can be attributed to the energy demand associated with data manipulation. In the capture phase (green), the device consumes 8.5 mA, which reflects the additional power requirements associated with data acquisition. The saving phase (orange) demonstrates a notable increase in energy consumption, reaching up to 53 mA, which is indicative of the substantial power required for write operations to the SD card. A comprehensive overview of these values can be found in Table 3. The mean consumption for a 32 MHz microcontroller clock frequency and sampling at 4000 Hz is 18 mA, which provides an estimated sensor autonomy of 12.2 h.

**Figure 4:**
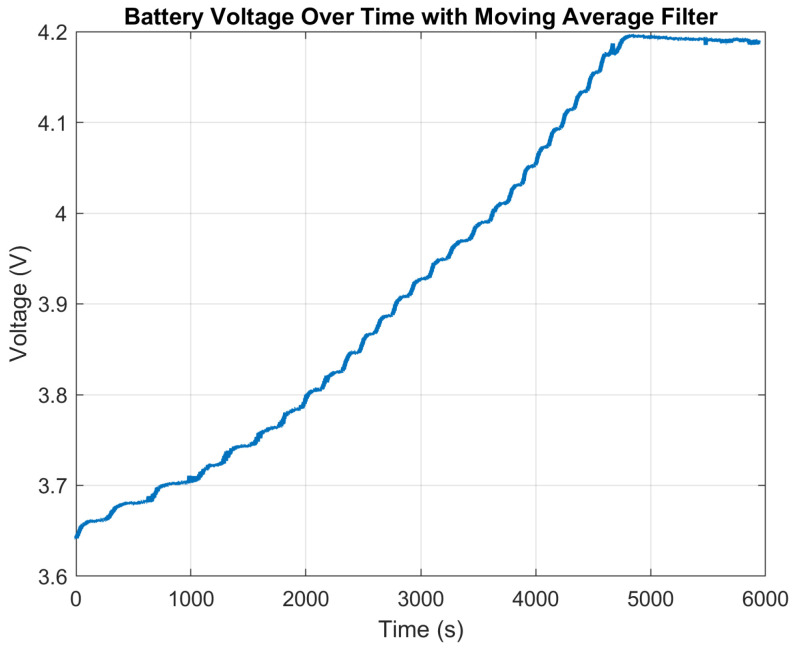
Battery charging.

The STNS01 battery charger is designed for single-cell batteries, using a CC-CV (Constant Current–Constant Voltage) algorithm for batteries up to 4.2 V. Charging starts in constant current mode and is adjustable up to 0.2 mA. Upon reaching the float voltage of 4.2 V, the charger shifts to constant voltage mode, maintaining the voltage and gradually reducing the charging current, as shown in Figure 4. The process ends when the current drops below 10% of IFAST, signaling the completion of the charge cycle.

**Figure 5:**
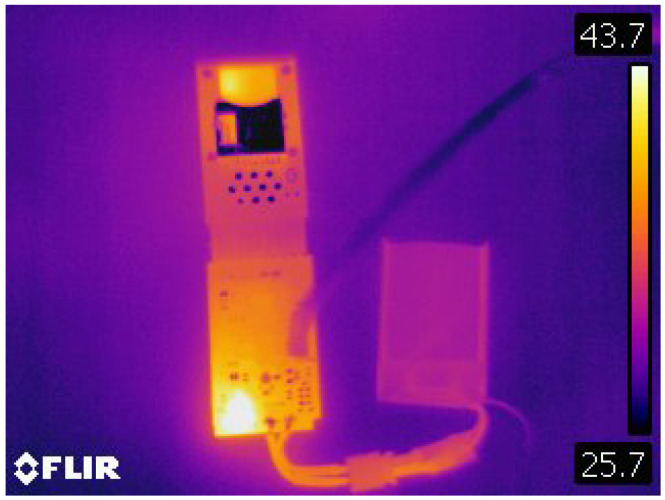
Thermal image of sensor charging process, highlighting the STNS01’s role as a BMS.

In the thermal image presented in Figure 5, the charging process of a sensor is observed, wherein the STNS01 chip functions as a Battery Management System (BMS). The thermographic profile clearly shows the STNS01 as the predominant heat source, achieving a maximum temperature of 43.7 °C. This temperature is within the expected and safe operational limits for the circuit.

### 3.3. Standard Deviation

**Figure 6:**
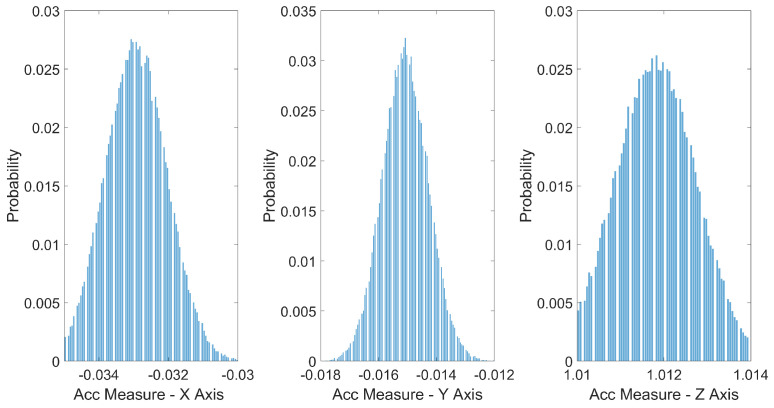
Standard deviation for accelerometer.

The obtained results reveal the standard deviations of the accelerometer in the X, Y, and Z axes (Figure 6), providing a quantitative measure of the noise level present in the measurements. The standard deviation in the X axis is 0.912 mg, that in the Y axis is 0.812 mg, and that in the Z axis is 0.956 mg. It is noteworthy that, despite the presence of noise, the results exhibit relatively low standard deviations.

Gyroscope data also indicate low variability in the measurements. The standard deviations in the X, Y, and Z axes of the gyroscope are 0.0931 dps, 0.4142 dps, and 0.2658 dps, respectively. This consistency in the gyroscope results complements the overall quality of the measurements.

### 3.4. Drift

**Figure 7:**
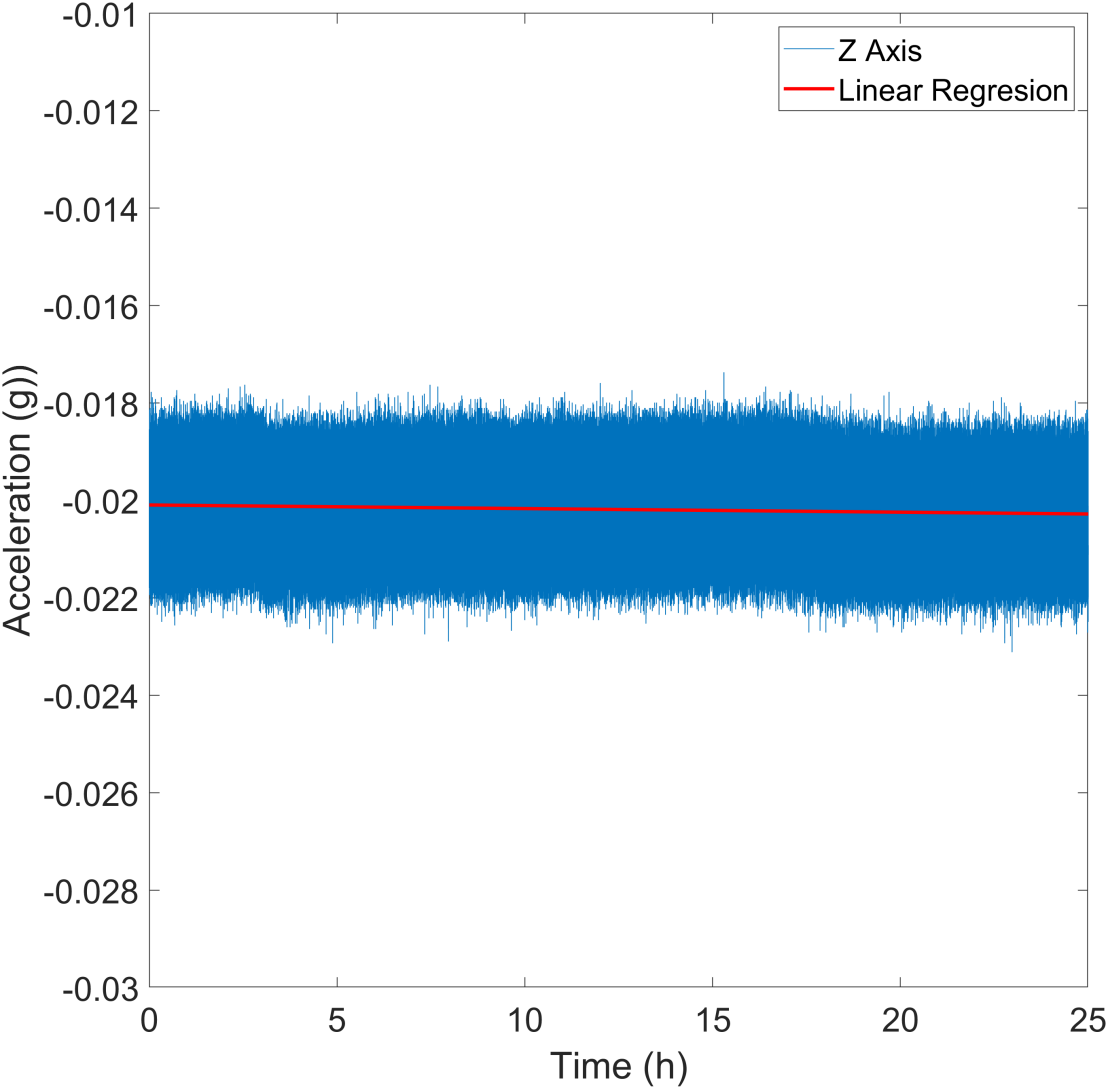
Accelerometer drift for x axis.

Following a continuous 24-h data collection period, the calculated drift values for the x (Figure 7), y, and z axes of the accelerometer are $-0.4523\times 10^{-6}$ g, $2.7690\times 10^{-6}$ g, and $0.2511\times 10^{-6}$ g, respectively. In the case of the gyroscope, the drift is $4.4976\times 10^{-5}$ dps for the X axis, $-4.5143\times 10^{-5}$ dps for the Y axis, and $2.1891\times 10^{-5}$ dps for the Z axis. These values are significantly lower than the sensitivity threshold; thus, they can be considered negligible.

### 3.5. Fixed Bias

**Figure 8:**
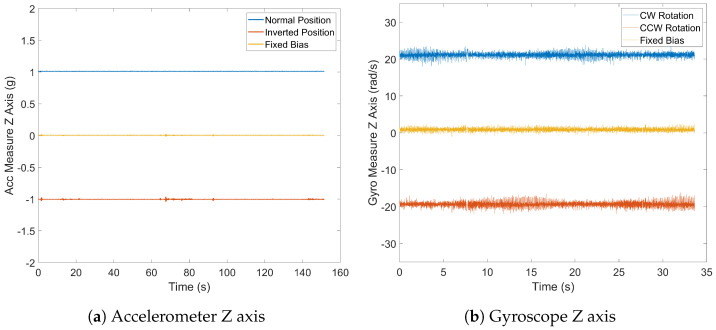
Fixed bias.

The fixed bias values for the accelerometer (Figure 8a) are notably close to zero, minimizing systematic errors. The mean fixed bias for the x axis is 4.33 mg, that for the y axis is −8.52 mg, and that for the z axis is 3.07 mg. These results suggest minimal systematic offset along each axis. Conversely, the gyroscope’s fixed bias results (Figure 8b) deviate further from the ideal zero values. The mean fixed bias along the x axis is 0.90840 dps, that along the y axis is −1.69095 dps, and that along the z axis is 0.02031 dps. These deviations may stem from manufacturing tolerances, sensor imperfections, or environmental conditions, introducing systematic errors in measurements and impacting the gyroscope’s accuracy.

### 3.6. Scale Factor

**Figure 9:**
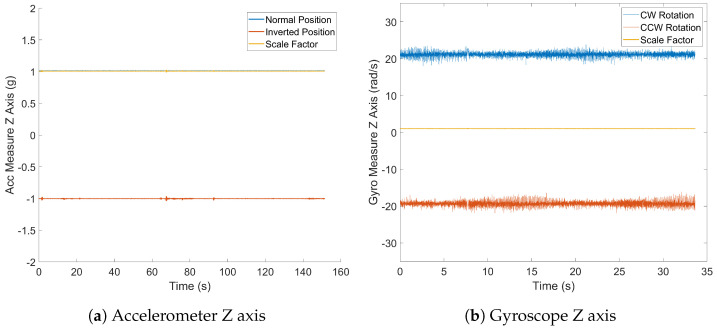
Scale factor.

The obtained scale factor results for both the accelerometer and gyroscope demonstrate exceptional performance and consistency. The accelerometer exhibits a mean scale factors close to the ideal value of 1:along the x axis of 1.00744, along the y axis of 0.99888, and along the z axis of 1.00100 (Figure 9) Similarly, the gyroscope’s mean scale factors, despite slightly deviating from 1, indicate remarkable consistency. Along the x axis, the scale factor is 1.01115, that along the y axis is 0.99969, and that along the z axis is 0.99201.

### 3.7. Power Spectral Density

**Figure 10:**
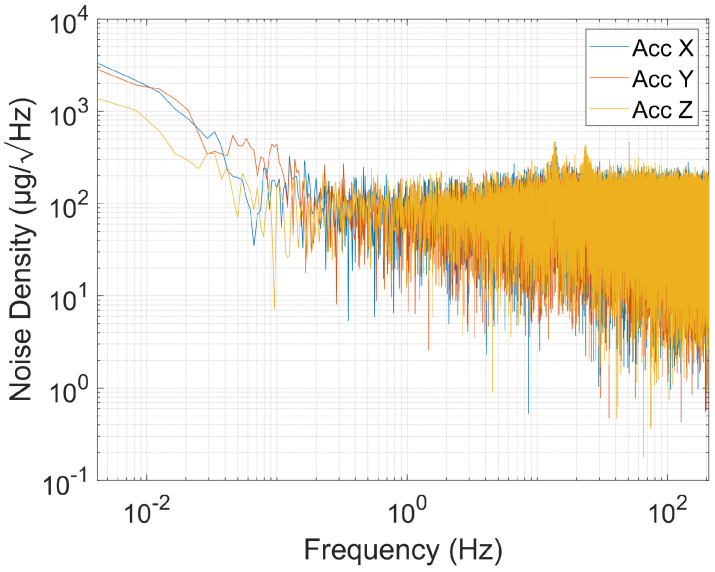
Accelerometer power spectral density.

Noise density plots in power spectral density (PSD) provide valuable insights into sensor performance, as illustrated in Figure 10 for accelerometer and gyroscope PSDs. The noise density in the accelerometer for the X and Y axes (Table 4) closely matches the datasheet specification of 80 µg/$\sqrt{\mathrm{Hz}}$ [12], except for the Z axis, which exhibits a significantly higher value. Conversely, the gyroscope’s power spectral density (PSD) for the Y and Z axes is approximately 20 mdps/$\sqrt{\mathrm{Hz}}$, compared to the specified 4 mdps/$\sqrt{\mathrm{Hz}}$ in the datasheet [12]. In this case, the X axis is the closest to the desired value, with a PSD of 7.26 mdps/$\sqrt{\mathrm{Hz}}$.

### 3.8. Allan Deviation

**Figure 11:**
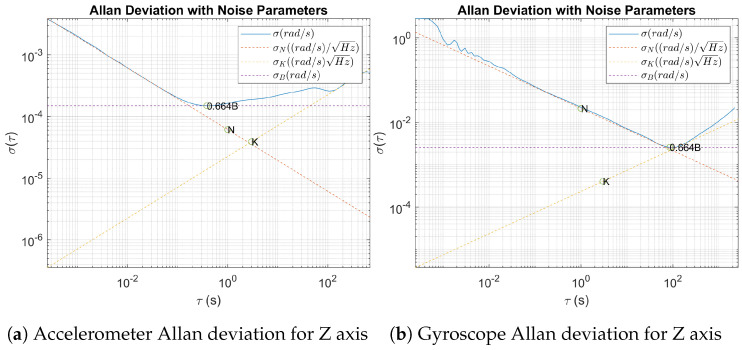
Allan deviation.

Allan deviation analysis of the accelerometer Figure 11a and the gyroscope reveals critical insights into its noise characteristics, highlighting Angular Random Walk, Bias Instability, and Rate Random Walk.

The Allan deviation plot (Figure 11b) for the Z axis provides valuable insights into the sensor’s noise characteristics. The blue line represents the Allan deviation over various integration times (τ). Initially, at short integration times, the plot exhibits a negative slope close to −0.5, indicating the presence of Angular Random Walk (ARW). The fitted red dashed line for ARW confirms this, with a slope of −0.5, suggesting that the dominant noise source at high frequencies is ARW, which is typical for white noise in gyroscope measurements. The ARW coefficient (N) is extracted and represented by point ‘N’ on the graph, showing the expected behavior of the gyroscope in the presence of random walk noise.

As the integration time increases, the plot transitions to a flatter region, as highlighted by the purple dashed line, indicating bias instability (B). This flat region signifies that the noise is dominated by a constant bias drift over time. The bias instability coefficient (0.664B) is identified, representing the minimal variance achieved over the specified τ. At longer integration times, the plot exhibits a positive slope close to 0.5, suggesting the presence of Rate Random Walk (RRW). The yellow dashed line fitted for RRW confirms this behavior, indicating that low-frequency noise begins to dominate, leading to a divergence in the Allan deviation. The coefficient (K) for RRW is also extracted, providing a comprehensive understanding of the gyroscope’s noise performance across different time scales. This analysis is crucial for applications requiring precise gyroscopic measurements over varying durations, as it helps in identifying and mitigating the impact of different noise sources.

## 4. Discussion

The experiments indicate that the sampling frequency is contingent upon two key factors, namely the SPI interface and the processing time of the microcontroller. While the sensor was engineered to possess superior sampling capabilities when compared to sensors currently on the market, future iterations could benefit from modifications in these two aspects in order to enhance the overall optimization of the device.

The power consumption analysis reveals that the primary peaks occur during the processes of opening and saving data to the SD card. It can be seen, therefore, that achieving optimal energy management is closely linked to the size of the acquisition buffer storage, as larger buffers can reduce the frequency of these high-power operations. This suggests that by adjusting the buffer size correctly, the number of times the system needs to access the memory can be reduced, thereby enhancing overall energy efficiency.

The PSD results show discrepancies that may arise from variations in calibration during production, differences in environmental conditions during testing, or potential measurement errors. It is essential to understand these factors in order to conduct a comprehensive evaluation of the sensor’s actual performance.

In light of the findings, it is advised that three additional refinements be made to the design. First, the sensor–controller connection should utilize a dedicated SPI interface, while the SD card should be connected via the SDIO interface. This will enhance storage speed and eliminate potential bottlenecks in the sampling and storage process. Second, replacing the BMS components with an LDO and using the microcontroller for battery management will reduce power consumption and heat generation from the current BMS components, which can affect the IMU results. Finally, eliminating the SD socket will save space and costs while increasing the device’s hermeticity.

A comparison of our design, named Bimu, with other similar devices on the market, including the NilsPod (Portabiles, Erlangen, Germany), Physilog^®^5 (Gait Up, Lausanne, Switzerland), Shimmer 3 (Shimmer Research, Dublin, Ireland), and XSens Dot (Movella, Henderson, NV, USA) is presented in Table 5, revealing a number of distinctive features. Bimu is a compact device, with competitive dimensions of 45.5 × 24 × 9.7 mm. It boasts a substantial 32 GB of onboard memory—the highest among the devices under review—which allows for the extensive collection of data without the necessity of data offloading. Furthermore, Bimu has the second-highest battery capacity of 220 mAh and a maximum sampling rate of 4000 Hz, enabling detailed, high-fidelity data acquisition, which is crucial for event-driven motion analysis. Additionally, while some devices feature integrated sensor fusion, Bimu and Portabiles require post-processing. Bimu outputs raw signals, with no on-sensor signal processing. However, this limitation is potentially addressable through future firmware updates, alongside the incorporation of additional functionalities like gait or balance analysis.

## 5. Conclusions

This study presents the design and characterization of a wearable IMU device with potential applications in health care and sports. The device performs well in terms of energy efficiency and data storage but requires optimization in the areas of sensor connectivity and battery management. Through noise analysis, it was identified that there is low variability in the majority of axes; however, discrepancies in the Z axis warrant further investigation. Bias measurements indicated minimal errors in the accelerometer, although the gyroscope showed more significant deviations. Scale factor tests confirmed the sensor’s reliability, with results close to ideal values.

For researchers working in similar domains, our findings highlight the importance of optimizing sensor characteristics to meet specific application needs. The detailed evaluation of noise, bias, and scale factors in this study is directly relevant to those interested in developing or refining wearable IMUs. As the wearable technology market continues to expand, future research should build on these insights, exploring further enhancements to improve device performance and adaptability across a broader range of applications.

## Figures and Tables

**Table 1:** Comparison of IMU sensors.

Specification	LSM6DSL	MPU6050	BMI270	ICM-42670-P
Acc Range (g)	±2 to ±16	±2 to ±16	±2 to ±16	±2 to ±16
Acc Sensitivity (mg/LSB)	0.061 to 0.488	0.061 to 0.488	0.061 to 0.488	0.061 to 0.488
Gyro Range (dps)	±125 to ±2000	±250 to ±2000	±125 to ±2000	±250 to ±2000
Gyro Sensitivity (mdps/LSB)	4.375 to 70	7.63 to 61	3.81 to 61	7.63 to 61
Magnetometer Range (gauss)	N/A	N/A	N/A	±4 to ±16
Power Consumption (mA)	0.65 (Acc + Gyro)	3.9 (Acc + Gyro)	0.685 (Acc + Gyro)	0.55 (Acc + Gyro)
Sampling Frequency (Hz)	12.5 to 6600	4 to 8000	12.5 to 1600	12.5 to 1600

**Table 2:** Comparison of microcontroller families.

Specification	STM32L051	ESP32-S3	AT32UC3	Atxmega32C4
Max Frequency [MHz]	32	240	50	32
Flash Memory [KBytes]	32	384	32	32
RAM [KBytes]	8	512	16	4
ADC	16-channel	20-channel	8-channel	16-channel
	12-bit	12-bit	12 bit	12-bit
Active Power [mA]	4.4	66.7	8.3	7.5
Idle Power [mA]	1.2	32.9	2.9	2.7
Sleep Power [uA]	0.29	8	4.7	1
Other		BlueTooth 5		
		WiFi		

**Table 3:** Power consume.

Idle phase	5.9 mA
File initialization phase	44 mA
Measurement phase	8.5 mA
Save phase	53 mA
Average consume	18 mA

**Table 4:** PSD.

	Accelerometer			Gyroscope	
X Axis	Y Axis	Z Axis	X Axis	Y Axis	Z Axis
86.865340 µg/$\sqrt{\mathrm{Hz}}$	72.835314 µg/$\sqrt{\mathrm{Hz}}$	395.418727 µg/$\sqrt{\mathrm{Hz}}$	7.259788 mdps/$\sqrt{\mathrm{Hz}}$	35.164361 mdps/$\sqrt{\mathrm{Hz}}$	22.681209 mdps/$\sqrt{\mathrm{Hz}}$

**Table 5:** Comparison with other commercial devices.

Specifications	Bimu	Portabiles	Gait Up	Shimmer	Xsens Dot
Dimensions (mm)	45.5 × 24 × 9.7	28 × 23 × 11.5	47.5 × 26.5 × 10	51 × 34 × 14	36.3 × 30.35 × 10.85
Onboard Memory	32 GB	250 MB	8 GB	8 GB	-
Battery Capacity	220 mAh	120 mAh	140 mAh	450 mAh	70 mAh
Max. Sampling Rate	4000 Hz	1024 Hz	512 Hz	1024 Hz	120 Hz
Accelerometer Range (±16 g)	●	●	●	●	●
Gyroscope Range (±2000 deg/s)	●	●	●	●	●
		Temperature	Temperature	Altimeter	
Charging Options	Custom cable	Wireless	Micro USB	Micro USB/Dock	Micro USB/Dock
Waterproof			IP64		IP68
Developer Options	Python API		Python API	Java API	Xsens SDK
	Matlab Instrument Driver		Matlab Instrument Driver	Matlab Instrument Driver	xSens DOT App
			C#/C++ API	LabVIEW Instrument Driver	Xsens DOT Server
				C#/C++ API	
Raw Data	●	●	●	●	●
Onboard Sensor Fusion			●	●	●
Other Functionalities	Pedometer		Gait, balance, motor test		

## Data Availability

The data presented in this study are available upon request from the corresponding author.

## Abbreviations

The following abbreviations are used in this manuscript:

ADC	Analog-to-Digital Converter
ARW	Angular Random Walk
BMS	Battery Management System
FIFO	First In, First Out
IMU	Inertial Measurement Unit
LDO	Low Dropout Regulator
MDPI	Multidisciplinary Digital Publishing Institute
NTC	Negative Temperature Coefficient
PCB	Printed Circuit Board
PSD	Power Spectral Density
RAM	Random Access Memory
RRW	Rate Random Walk

## References

[1] Garcia-De-Villa S., Casillas-Perez D., Jimenez-Martin A., Garcia-Dominguez J.J. (2023). Inertial Sensors for Human Motion Analysis: A Comprehensive Review. IEEE Trans. Instrum. Meas..

[2] Friend S.H., Ginsburg G.S., Picard R.W. (2023). Wearable Digital Health Technology. N. Engl. J. Med..

[3] Nijmeijer E.M., Heuvelmans P., Bolt R., Gokeler A., Otten E., Benjaminse A. (2023). Concurrent validation of the Xsens IMU system of lower-body kinematics in jump-landing and change-of-direction tasks. J. Biomech..

[4] Gu C., Lin W., He X., Zhang L., Zhang M. (2023). IMU-based motion capture system for rehabilitation applications: A systematic review. Biomim. Intell. Robot..

[5] Digo E., Gastaldi L., Antonelli M., Pastorelli S., Cereatti A., Caruso M. (2022). Real-time estimation of upper limbs kinematics with IMUs during typical industrial gestures. Procedia Comput. Sci..

[6] Faisal A.I., Majumder S., Scott R., Mondal T., Cowan D., Deen M. (2021). A Simple, Low-Cost Multi-Sensor-Based Smart Wearable Knee Monitoring System. IEEE Sensors J..

[7] Hage R., Detrembleur C., Dierick F., Pitance L., Jojczyk L., Estievenart W., Buisseret F. (2020). DYSKIMOT: An Ultra-Low-Cost Inertial Sensor to Assess Head’s Rotational Kinematics in Adults during the Didren-Laser Test. Sensors.

[8] Patrizi G., Carratù M., Ciani L., Sommella P., Catelani M., Pietrosanto A. (2024). Temperature Sensitivity Analysis of Inertial Measurement Unit under Dynamic Conditions. IEEE Trans. Instrum. Meas..

[9] Ailneni S., Kashyap S.K., Kumar N.S., Livingstone D.D., Aishwarya N., Varghese N., Karthik K.V. Characterization of MEMS based Inertial Measurement Unit. Proceedings of the 1st International Conference on Range Technology, ICORT 2019.

[10] Zhang P., Zhan X., Zhang X., Zheng L. (2019). Error characteristics analysis and calibration testing for MEMS IMU gyroscope. Aerosp. Syst..

[11] (1998). IEEE Standard Specification Format Guide and Test Procedure for Single-Axis Interferometric Fiber Optic Gyros.

[12] STMicroelectronics (2023). LSM6DSL Datasheet.

[13] Invensense (2013). MPU6070 Datasheet.

[14] Bosh (2021). BMI270 Datasheet.

[15] Invensense (2021). ICM-42670-P Datasheet.

[16] STMicroelectronics (2023). STM32L051 Datasheet.

[17] Espressif Systems (2023). ESP32-S3 Series Datasheet.

[18] ATMEL (2012). AT32UC3L Series Datasheet.

[19] ATMEL (2014). ATxmega32C4 Series Datasheet.

